# Association of pneumonia admission with polypharmacy and drug use in community‐dwelling older people

**DOI:** 10.1111/ggi.14860

**Published:** 2024-03-18

**Authors:** Hironobu Hamaya, Taro Kojima, Yukari Hattori, Masahiro Akishita

**Affiliations:** ^1^ Department of Geriatrics Tokyo Metropolitan Institute for Geriatrics and Gerontology Tokyo Japan; ^2^ Department of Geriatric Medicine The University of Tokyo Tokyo Japan

**Keywords:** anticholinergics, pneumonia, polypharmacy, potentially inappropriate medication

## Abstract

**Aim:**

The purpose of the present study was to clarify the association of pneumonia admission with polypharmacy and specific drug use in community‐dwelling older people.

**Methods:**

Using health insurance and long‐term care insurance data from Kure city in Japan, we retrospectively collected data for older community‐dwelling people (aged ≥65 years) from April 2017 to March 2019. The outcome was pneumonia admission. We carried out multivariate logistic regression analysis to identify the association of pneumonia admission with polypharmacy (≥5 drugs), the use of psychotropic drugs or anticholinergics with adjustment for patient backgrounds, such as comorbidity, and the daily life independence level for the older people with disability.

**Results:**

Of 59 040 older people, 4017 (6.8%) participants were admitted for pneumonia in 2 years. The ratio of polypharmacy, and the use of psychotropic drugs and anticholinergics in the admission group were significantly higher than the non‐admission group. Multivariate logistic regression analysis showed that polypharmacy (odds ratio 1.29, 95% confidence interval 1.18–1.41), and the use of conventional antipsychotic drugs (odds ratio 1.39, 95% confidence interval 1.02–1.90), atypical antipsychotic drugs (odds ratio 1.67, 95% confidence interval 1.37–2.05) and anticholinergics (odds ratio 1.22, 95% confidence interval 1.13–1.33) were significantly associated with pneumonia admission.

**Conclusion:**

The present results suggest that polypharmacy, and the use of psychotropic drugs and anticholinergics are risk factors for pneumonia admission. **Geriatr Gerontol Int 2024; 24: 404–409**.

## Graphical abstract

The results showed that polypharmacy (odds ratio [OR] 1.29, 95% confidence interval [CI] 1.18–1.41), and the use of atypical antipsychotic drugs (OR 1.67, 95% CI 1.37–2.05) and anticholinergics (OR 1.22, 95% CI 1.13–1.33) were significantly associated with pneumonia admission in older community‐dwelling persons without disability (polypharmacy ≥5 drugs).

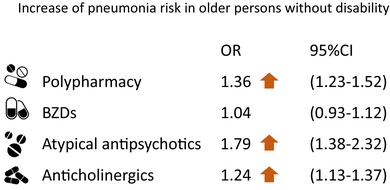

## Introduction

In an aging society, pneumonia has become an important health problem. In Japan, pneumonia is the fifth leading cause of death, and aspiration pneumonia is the sixth leading cause of death.^1^ When combined, it surpasses the fourth leading cause of death, cerebrovascular disease, and it is expected that many of the pathological conditions of pneumonia are included in the third leading cause of death, senility. Approximately 98% of deaths from pneumonia are in older people aged ≥65 years, and dysphagia has a major impact on pneumonia in older people. According to a report by Teramoto *et al*., the rate of aspiration pneumonia increased with age among patients admitted for pneumonia, and that approximately 80% of pneumonia patients aged ≥70 years had aspiration pneumonia.^2^

Protective mechanisms against dysphagia include upper airway reflexes (swallowing reflex and coughing reflex). Both reflexes decline due to age‐related changes, such as muscle atrophy and decreased smoothness involved in swallowing, changes in posture, such as hunched posture, and decreased cognitive function. In addition, substance P has been reported as a neurotransmitter involved in upper airway reflexes, and the use of drugs with antidopaminergic action were reported to reduce reflexes and the concentration of substance P substances.^3^, ^4^, ^5^ Thus, there is a close relationship between drug use and dysphagia.

In addition, the number of older people with multimorbidity and multiple geriatric syndromes is increasing, along with global aging, and as a result, older people are prone to polypharmacy.^6^, ^7^ Wastesson *et al*. reported a rapid increase in the prevalence of polypharmacy internationally over the decades, and polypharmacy has been reported to cause an increase in falls/fractures, deterioration in cognitive function, deterioration in physical function, and an increase in admission and institutionalization.^8^, ^9^, ^10^, ^11^, ^12^, ^13^, ^14^ If polypharmacy is associated with dysphagia, it might be a risk factor for pneumonia, but this has not been established.

Thus, the present study investigated the association of polypharmacy, drug use and pneumonia admission, especially the influence of psychotropic drugs or anticholinergics.

## Methods

The present study was a population‐based retrospective observational study using health insurance and long‐term care insurance data from Kure city in Japan from April 2017 to March 2019. Data for analysis were extracted from an anonymized health insurance and long‐term care insurance claims database of citizens aged ≥65 years living in Kure city, Japan. These were obtained from the Data Horizon Corporation with approval from Kure city, which manages the data. A total of 67 169 individuals were included in this study. Exclusion criteria were as follows: (1) died during the observation period (*n* = 4605); and (2) missing data difficult to follow up (*n* = 3524). Following these criteria, we analyzed the 59 040 eligible participants in the present study. The health insurance data included age, sex, major comorbidities (hypertension, dyslipidemia, diabetes mellitus, osteoporosis, chronic heart failure, Parkinson's disease, cerebral infarction, cardiac infarction, bronchial asthma and chronic obstructive pulmonary disease), and number and types of prescribed drugs at April 2017. The long‐term care insurance data included the daily life independence level for the older people with disability. Polypharmacy was defined as taking five or more prescribed drugs, as polypharmacy is widely accepted as taking five or more drugs in clinical studes.^15^ Psychotropic drugs were defined as benzodiazepine, non‐benzodiazepine sedative hypnotics, selective serotonin reuptake inhibitor, serotonin noradrenalin reuptake inhibitor, conventional antipsychotic drugs, atypical antipsychotic drugs and anticonvulsants. Anticholinergic drugs were defined as those included in the anticholinergic cognitive burden scale (ACB scale).^16^ The ACB scale is a list of drugs with possible effects on cognitive function. A multidisciplinary panel assessed individual drugs to have none, possible or definite anticholinergic properties, with a score ranging from 0 to 3. The ACB scale reported 99 medicines with known anticholinergic activity. Studies that used the ACB scale have shown that higher anticholinergic burden predicts cognitive impairment in older people.^17^, ^18^ The daily life independence level for the older people with disability was defined for the long‐term care insurance system in Japan,^19^ and is known to correlate closely with the globally applied Functional Independence Measure.^20^ The disability levels were categorized according to this daily life independence level using three levels: independent (rank J), semi‐bedridden (rank A) and bedridden (rank B and C). The primary outcome was admission for pneumonia from April 2017 to March 2019. ICD‐10 code J13‐18, J69 was used for the definition of pneumonia.

### Statistical analysis

Continuous variables are expressed as the mean ± standard deviation and percentage, as appropriate. Categorical variables are presented as the number and percentages. Student's *t*‐test was used to compare the averages of continuous variables (such as age), and the χ^2^‐test was used to compare the proportions of categorical variables (such as sex). The prescription ratio of the target drugs was also evaluated using the χ^2^‐test. A multivariate logistic regression analysis was carried out to evaluate the association between polypharmacy, the use of psychotropic drugs, anticholinergic drugs, ACB scale score and admission for pneumonia. Subgroup analyses were also carried out with participants stratified by daily life independence levels for older people with or without disability (independent, semi‐bedridden, bedridden). All factors were included in multivariate logistic regression analysis. Values of *P* < 0.05 were considered statistically significant. Data were analyzed using spss version 25.0 (IBM, Armonk, NY, USA).

### Ethical consideration

This study was approved by the Ethical Committee of The University of Tokyo (No. 2019004NI). The need for written informed consent was waived due to this being a retrospective anonymized database analysis study.

## Results

**Figure 1:**
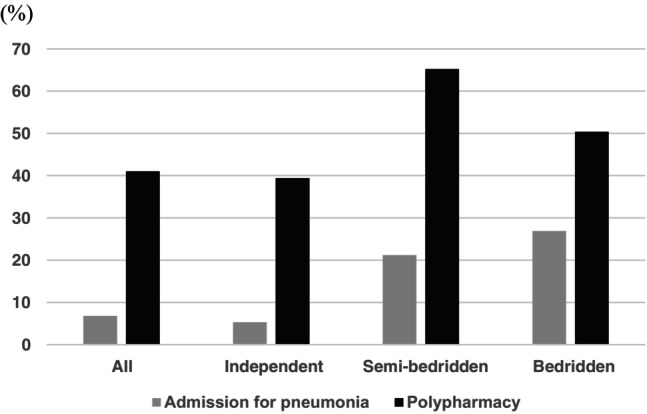
The frequency of admission for pneumonia and polypharmacy by the disability levels.

The total number of participants for the analysis was 59 040. Table 1 shows the characteristics of participants with admission for pneumonia and non‐ admission for pneumonia. A total of 4017 individuals (6.8%) were identified who were admitted to the hospital for pneumonia during the observational period (2017–2019). Compared with the non‐admission group, the admission group was older (75.9 ± 7.2 vs 81.9 ± 7.6, *P* < 0.0001), more were men (39.7% vs 41.9%, *P* = 0.006) and they had many diseases. The rate of polypharmacy (number of drugs ≥5) was also higher in the admission group (40.0% vs 56.8%, *P* < 0.0001), and they had a greater number of prescriptions for psychotropic drugs, which are benzodiazepines, non‐benzodiazepines sedative hypnotics, selective serotonin reuptake inhibitors, serotonin noradrenalin reuptake inhibitors and anticonvulsants. The prescription of anticholinergic drugs was also significantly prevalent in the admission group (20.2% vs 33.2%, *P* < 0.001). The prevalence of admission for pneumonia and polypharmacy were 5.3% and 39.4% in the independent group, 21.2% and 65.2% in the semi‐bedridden group, and 26.9% and 50.4% in the bedridden group, respectively (Fig. 1).

**Table 1:** Characteristics of participants by admission or non‐admission by pneumonia in 2 years (2017–2019)

	Non‐admission group	Admission group	
Factors	*n* = 55 023	*n* = 4017	*P*‐value
Men	39.7%	41.9%	0.006
Age (years)	75.9 ± 7.2	81.9 ± 7.6	<0.001
Age group (years)			
65–74	46.5%	17.8%	<0.001
75–84	40.1%	42.6%	
85–94	12.5%	35.7%	
≥95	0.9%	3.9%	
Disability			<0.001
Independent	93.2%	71.8%	
Semi‐bedridden	4.3%	16.0%	
Bedridden	2.4%	12.2%	
Hypertension	54.1%	59.3%	<0.001
Dyslipidemia	42.4%	34.7%	<0.001
Diabetes mellitus	33.8%	35.6%	<0.05
Osteoporosis	18.4%	25.9%	<0.001
Chronic heart failure	17.9%	31.0%	<0.001
Parkinson's disease	1.1%	3.4%	<0.001
Cerebral infarction	9.1%	13.0%	<0.001
Myocardial infarction	1.9%	3.3%	<0.001
Bronchial asthma	9.1%	13.5%	<0.001
COPD	7.1%	14.8%	<0.001
Dementia	5.4%	19.2%	<0.001
No. drugs	4.1 ± 3.8	5.6 ± 4.4	<0.001
Polypharmacy (no. drugs ≥5)	40.0%	56.8%	<0.001
Benzodiazepines	12.8%	17.5%	<0.001
Non‐benzodiazepines	5.1%	6.2%	0.001
SSRIs	1.2%	1.7%	0.003
SNRIs	0.9%	1.7%	<0.001
Conventional antipsychotic drugs	0.5%	1.4%	<0.001
Atypical antipsychotic drugs	1.0%	4.1%	<0.001
Anticonvulsants	2.4%	5.2%	<0.001
Anticholinergic agents	20.2%	33.2%	<0.001

*Note*: Total *n* = 59 040. Data are presented as mean ± standard deviation or percentage within each group. Student's *t‐*test or χ^2^‐test were used for the analysis.

Abbreviations: COPD, chronic obstructive pulmonary disease; SNRI, serotonin noradrenalin reuptake inhibitor; SSRI, selective serotonin reuptake inhibitor.

Table 2 shows the prevalence of prescription of psychotropic drugs and anticholinergic drugs in the polypharmacy or non‐polypharmacy group. The prescriptions for each psychotropic drug and anticholinergic drug were significantly higher in the polypharmacy group.

**Table 2:** Prescription of psychotropic drugs and anticholinergic drugs in the polypharmacy group and non‐polypharmacy group

Factors	Polypharmacy (*n* = 24 229)	Non‐polypharmacy (*n* = 34 811)	*P*‐value
Benzodiazepine	25.2%	4.7%	<0.001
Non‐benzodiazepines	9.8%	2.0%	<0.001
SSRIs	2.4%	0.4%	<0.001
SNRIs	2.1%	0.2%	<0.001
Conventional antipsychotic drugs	1.3%	0.1%	<0.001
Atypical antipsychotic drugs	2.5%	0.4%	<0.001
Anticonvulsants	5.1%	0.8%	<0.001
Anticholinergic drugs	40.2%	7.7%	<0.001

*Note*: Data are presented as the percentage within each group.

Abbreviations: SNRI, serotonin noradrenalin reuptake inhibitor; SSRI, selective serotonin reuptake inhibitor.

The proportion of comorbidities by disabilities are shown in Table S1. The prevalence of Parkinson's disease was 0.9%, 4.2% and 4.5% in the independent group, semi‐bedridden group and bedridden group, respectively. Accordingly, the prevalence of stroke was 8.8%, 17.3% and 12.4% in the independent group, semi‐bedridden group and bedridden group, respectively. On multivariate logistic regression analysis (Table 3), the admission for pneumonia was significantly associated with older age group and diseases, such as osteoporosis, chronic heart failure, Parkinson's disease, cerebral infarction, myocardial infarction, bronchial asthma, chronic obstructive pulmonary disease and dementia. Polypharmacy and use of drugs, such as serotonin noradrenalin reuptake inhibitor, conventional antipsychotic drugs, atypical antipsychotic drugs, anticonvulsants and also anticholinergic drugs, were significantly associated with admission for pneumonia. To check whether disability levels (assessed by the daily life independence levels for the older people) affect these risks, subgroup analysis was carried out (Table 4). In the independent group, polypharmacy, atypical antipsychotic drugs and anticholinergic drugs were significantly associated with admission for pneumonia. However, in the semi‐bedridden group, polypharmacy was not associated with admission for pneumonia. In the bedridden group, non‐polypharmacy was significantly associated with admission for pneumonia. These results showed that there were differences in the relationship of specific drugs and having pneumonia.

**Table 3:** Logistic regression analysis of the association of admission for pneumonia with polypharmacy, and use of psychotropic and anticholinergic drugs

	Univariate	Multivariate
Factors	OR (95% CI)	OR (95% CI)
Men	1.10 (1.03–1.17)	1.42 (1.32–1.53) **
Age group (years)		
65–74	Reference	Reference
75–84	2.78 (2.54–3.04)	2.36 (2.15–2.58)**
85–94	7.51 (6.83–8.24)	5.60 (5.06–6.20)**
≥95	12.16 (10.01–14.78)	9.12 (7.43–11.20)**
Hypertension	1.24 (1.16–1.32)	0.82 (0.75–0.88)
Dyslipidemia	0.72 (0.68–0.77)	0.68 (0.63–0.74)**
Diabetes mellitus	1.08 (1.01–1.16)	1.05 (0.97–1.13)
Osteoporosis	1.56 (1.45–1.68)	1.14 (1.05–1.24)*
Chronic heart failure	2.07 (1.93–2.22)	1.28 (1.18–1.40)**
Parkinson's disease	3.24 (2.69–3.92)	1.90 (1.55–2.34)**
Cerebral infarction	1.49 (1.36–1.64)	1.11 (1.00–1.23)*
Myocardial infarction	1.48 (1.37–1.59)	1.24 (1.02–1.51)*
Bronchial asthma	1.56 (1.42–1.72)	1.18 (1.06–1.31)*
COPD	2.27 (2.07–2.50)	1.60 (1.45–1.77)**
Dementia	4.13 (3.78–4.50)	2.07 (1.88–2.28)**
Polypharmacy (≥5)	1.98 (1.86–2.11)	1.29 (1.18–1.41)**
Benzodiazepines	1.45 (1.33–1.58)	0.98 (0.89–1.08)
Non‐benzodiazepines	1.25 (1.09–1.42)	0.93 (0.81–1.07)
SSRIs	1.46 (1.14–1.88)	0.98 (0.75–1.28)
SNRIs	1.92 (1.49–2.47)	1.34 (1.02–1.90)*
Conventional antipsychotic drugs	2.65 (1.99–3.53)	1.39 (1.02–1.90)*
Atypical antipsychotic drugs	4.09 (3.43–4.88)	1.67 (1.37–2.05)**
Anticonvulsants	2.25 (1.94–2.62)	1.50 (1.27–1.77)**
Anticholinergic drugs	1.97 (1.83–2.11)	1.22 (1.13–1.33)**

Abbreviations: CI, confidence interval; COPD, chronic obstructive pulmonary disease; OR, odds ratio; SNRI, serotonin noradrenalin reuptake inhibitor; SSRI, selective serotonin reuptake inhibitor.

* *P*‐value <0.05;

** *P*‐value <0.001.

**Table 4:** Multivariate logistic regression analysis of association of admission for pneumonia with polypharmacy, and use of psychotropic and anticholinergic drugs by the daily life independence levels for the older people (independent, semi‐bedridden, bedridden)

	Independent	Semi‐bedridden	Bedridden
Factors	OR (95% CI)	OR (95% CI)	OR (95% CI)
Polypharmacy (≥5)	1.36 (1.23–1.52)**	1.05 (0.82–1.35)	0.70 (0.51–0.95)*
Benzodiazepines	1.04 (0.93–1.16)	0.88 (0.69–1.13)	0.88 (0.63–1.23)
Non‐benzodiazepines	0.95 (0.81–1.12)	0.90 (0.64–1.26)	0.85 (0.51–1.44)
SSRIs	1.04 (0.76–1.41)	0.68 (0.34–1.38)	1.08 (0.50–2.32)
SNRIs	1.24 (0.89–1.73)	1.33 (0.74–2.39)	1.67 (0.78–3.59)
Conventional antipsychotic drugs	1.03 (0.67–1.58)	2.40 (1.20–4.80) *	2.05 (0.99–4.26)
Atypical antipsychotic drugs	1.79 (1.38–2.32)**	1.51 (0.98–2.32)	1.09 (0.67–1.77)
Anticonvulsants	1.46 (1.19–1.79)**	1.09 (0.75–1.59)	1.06 (0.67–1.67)
Anticholinergic drugs	1.24 (1.13–1.37)**	1.20 (0.97–1.49)	1.08 (0.81–1.44)

*Note*: Odds ratios and their 95% confidence intervals were calculated by each daily life independence level, adjusted for sex, age and comorbidity.

Abbreviations: CI, confidence interval; OR, odds ratio; SNRI, serotonin noradrenalin reuptake inhibitor; SSRI, selective serotonin reuptake inhibitor.

* *P*‐value <0.05;

** *P*‐value <0.001.

## Discussion

The present study used both health insurance and long‐term care insurance data to examine whether there was a relationship of pneumonia admission to polypharmacy and the use of specific drugs by adjusting for older peoples' background, comorbidities and degree of disability. A significant association was found between polypharmacy and pneumonia admission, but not in the semi‐bedridden group, and in the bedridden group, non‐polypharmacy was significantly associated with pneumonia admission. There are few reports that tried to clarify the association between polypharmacy and pneumonia. Some recent reports showed the relationship between polypharmacy and pneumonia, but did not evaluate frailty or disability.^21^, ^22^ In this regard, we consider the present report to be novel. Previous studies have reported the association of the use of benzodiazepines, antipsychotics and anticholinergics with pneumonia.^23^, ^24^, ^25^, ^26^, ^27^, ^28^, ^29^ There are also reports that polypharmacy increases the risk of adverse drug events and increases the risk of admission.

Compared with the non‐polypharmacy group, the polypharmacy group were prescribed more anticholinergic drugs and antipsychotic drugs that have been previously reported as a risk for pneumonia, but it was interesting to discover that there was a significant association with pneumonia admission and polypharmacy even when these drugs were added to the confounding factors.

The causes of polypharmacy associated with pneumonia admission include: (1) an increased risk of adverse drug events and drug–drug interactions, and (2) drugs that might directly or indirectly affect swallowing function. An increased number of drugs, or polypharmacy, has been reported to be a significant risk factor for adverse drug events.^9^, ^10^, ^11^, ^12^, ^13^, ^14^ It has been reported that 80% of older people with polypharmacy have at least one drug interaction through cytochrome P450.^30^ It has also been reported that the administration of drugs that can cause drug interactions is significantly associated with the risk of admission due to adverse drug events.^31^

In the present results, the polypharmacy group took more drugs related to dysphagia, such as psychotropic drugs and anticholinergic drugs, so it is possible that these adverse drug events occurred more strongly. However, frequently used drugs that do not directly influence swallowing ability could be a cause for pneumonia. For example, Ca channel antagonists and gastric acid suppressants are frequently prescribed to older patients,^32^ but Ca channel antagonists have been reported to relax the lower esophageal sphincter,^33^, ^34^ and gastric acid suppressants have been reported to reduce the bactericidal action of gastric acid, resulting in changes in the composition of intestinal bacteria and an increase in oral flora.^35^, ^36^, ^37^

When older people with constipation and gastroesophageal reflux use Ca channel antagonists, proton‐pump inhibitors or both together, the risk of reflux of bacteria‐rich gastric fluid into the oral cavity might increase, and the risk for developing pneumonia might also increase as a consequence. Accordingly, the use of drugs that potentially suppress swallowing function is associated with increased number of drugs, and polypharmacy, together with geriatric conditions, might increase the risk of pneumonia admission.

The interesting point in the present study was that there was no significant association between polypharmacy and pneumonia admission in both the semi‐bedridden and the bedridden groups.

Chen *et al*. reported no significant association between hospitalization for community‐acquired pneumonia and polypharmacy. The average Barthel Index of the participants was approximately 10, and 60% of the participants used nasogastric tubes. This is a similar population to our sub‐bedridden and bedridden groups, and the results are consistent with our report.^38^

The semi‐bedridden group and bedridden group were likely to have a high frequency of impaired swallowing function associated with comorbid diseases, and their effects might be stronger than drug‐induced dysphagia, and, thus, there was no significant association between polypharmacy and admission for pneumonia. In fact, comorbidities known to be a risk for pneumonia, such as Parkinson's disease, stroke and dementia, were more common in the semi‐bedridden and bedridden groups. In addition, the bedridden group might have had severe conditions; or physicians had reduced drugs in older patients with disability and with limited life expectancy due to inefficiency of drug therapies in these populations.^39^, ^40^

The limitations in the present study are as follows. First, because the present research is based on health insurance and long‐term care insurance databases, the presence or absence of diseases might not be accurate compared with clinical database studies. Next, as the severity of the disease was not assessed, the needs for combination therapy in older people were not assessed in the analysis. Additionally, we excluded participants who died or had missing data. There were possibilities that they could have affected the data. Finally, the drugs data were obtained only at the baseline, which implies that changes of prescription and medication adherence were not considered.

In conclusion, the present study found that polypharmacy was significantly associated with admission for pneumonia. The risk of admission for pneumonia due to polypharmacy is higher in independent older people, so it is important to review medications regularly. It would be important to examine whether the incidence of pneumonia could be decreased by reducing specific drugs or resolving polypharmacy.

## Disclosure statement

TK received honoraria from Pfizer. MA received research grants from Bayer Health Care, Chugai Pharmaceutical, Daiichi Sankyo, Eisai, Kracie Pharma, Mitsubishi‐Tanabe Pharm, Ono Pharmaceutical, Takeda Pharmaceutical and Tsumura, and honoraria from Daiichi Sankyo, Toa Eiyo and Towa Pharmaceutical. The other authors declare no conflict of interest.

## Supporting information


**Supplementary Table S1.** The frequency of comorbidities by the disability levels.

## Data Availability

This data is managed by Data Horizon Corporation Ltd under a contract with Kure City and is not available.
