# Supplementary material for: Association of pneumonia admission with polypharmacy and drug use in community‐dwelling older people
Source: Geriatr Gerontol Int. 2024 Mar 18;24(4):404–9. doi: 10.1111/ggi.14860 (PMC11503537; doi:10.1111/ggi.14860)
Supplement: Supplementary file 1 — Supplementary Table S1. The frequency of comorbidities by the disability levels. [file GGI-24-404-s001.docx]

Supplementary table 1. The frequency of comorbidities by the disability levels.

|  | Independence (N=54,190) | semi-bedridden (N=3035) | bedridden (N=1815) |
| --- | --- | --- | --- |
| Hypertension | 53.8% | 67.0% | 51.8% |
| Dyslipidemia | 42.5% | 39.9% | 27.5% |
| Diabetes mellitus | 33.8% | 38.6% | 30.5% |
| Osteoporosis | 17.8% | 34.4% | 25.8% |
| Chronic heart failure | 17.4% | 36.6% | 30.4% |
| Parkinson's disease | 0.9% | 4.2% | 4.5% |
| Cerebral infarction | 8.8% | 17.3% | 12.4% |
| Myocardial infarction | 1.9% | 3.3% | 1.8% |
| Bronchial asthma | 9.3% | 11.6% | 9.5% |
| COPD | 7.3% | 11.8% | 10.0% |
| Dementia | 4.4% | 26.8% | 30.2% |

Data are presented as percentage within each group.
